# Functional expression of TRPM7 as a Ca^2+^ influx pathway in adipocytes

**DOI:** 10.14814/phy2.14272

**Published:** 2019-10-24

**Authors:** Hana Inoue, Masato Inazu, Masato Konishi, Utako Yokoyama

**Affiliations:** ^1^ Department of Physiology Tokyo Medical University Tokyo Japan; ^2^ Institute of Medical Science Tokyo Medical University Tokyo Japan

**Keywords:** Adipocyte, calcium pathway, TRP channel, TRPM7

## Abstract

In adipocytes, intracellular Ca^2+^ and Mg^2+^ modulates physiological functions, such as insulin action and the secretion of adipokines. TRPM7 is a Ca^2+^/Mg^2+^‐permeable non‐selective cation channel. TRPM7 mRNA is highly expressed in adipose tissue, however, its functional expression in adipocytes remains to be elucidated. In this study, we demonstrated for the first time that TRPM7 was functionally expressed in both freshly isolated white adipocytes and in 3T3‐L1 adipocytes differentiated from a 3T3‐L1 pre‐adipocyte cell line by whole‐cell patch‐clamp recordings. Consistent with known properties of TRPM7 current, the current in adipocytes was activated by the elimination of extracellular divalent cations and the reduction of intracellular free Mg^2+^ concentrations, and was inhibited by the TRPM7 inhibitors, 2‐aminoethyl diphenylborinate (2‐APB), hydrogen peroxide (H_2_O_2_), N‐methyl maleimide (NMM), NS8593, and 2‐amino‐2‐[2‐(4‐octylphenyl)ethyl]‐1,3‐propanediol (FTY720). Treatment with small‐interfering (si) RNA targeting TRPM7 resulted in a reduction in the current to 23 ± 7% of nontargeting siRNA‐treated adipocytes. Moreover a TRPM7 activator, naltriben, increased the TRPM7‐like current and [Ca^2+^]_i_ in 3T3‐L1 adipocytes but not in TRPM7‐knockdown adipocytes. These findings indicate that TRPM7 is functionally expressed, and plays a role as a Ca^2+^ influx pathway in adipocytes.

## Introduction

Transient receptor potential (TRP) proteins are a superfamily of cation channels, most of which are Ca^2+^‐permeable and thereby involved in Ca^2+^‐dependent signaling pathways. TRP melastatin (TRPM) 7 channel permeates Ca^2+^ and Mg^2+^, and is expressed in most cell types (Nadler et al. 2001; Runnels et al. 2001; Fonfria et al. 2006; Kunert‐Keil et al. 2006). TRPM7 is a very unique membrane protein in that it is a channel harboring an alpha‐kinase at the carboxyl‐terminal. Down‐regulation of TRPM7 in cells has been reported to affect diverse physiological functions, including cell proliferation, survival, and migration (reviewed in Bates‐Withers et al. 2011; Fleig and Chubanov 2014).

In adult humans, TRPM7 mRNA is most highly expressed in adipose tissue (Fonfria et al. 2006). In pre‐adipocytes, it has been demonstrated that the pharmacological blockade or siRNA of TRPM7 inhibited proliferation and adipogenesis via reduction in the expression of cyclin D1 and cyclin E kinases, and phosphorylated ERK1/2 and Akt (Chen et al. 2014). In contrast, it remains unclear whether TRPM7 is functionally expressed and plays physiological roles in mature adipocytes. It has been reported in mature adipocytes that [Ca^2+^]_i_ affects insulin signaling (Draznin et al. 1987; Kelly et al. 1989; Whitehead et al. 2001; Worrall and Olefsky 2002), lipolysis (Tebar et al. 1996; Xue et al. 1998), and the secretion of adipokines, including leptin (Levy et al. 2000; Cammisotto and Bukowiecki 2004), adiponectin (Sukumar et al. 2012; Komai et al. 2014; El Hachmane et al. 2015), and fatty acid binding protein 4 (FABP4) (Schlottmann et al. 2014). Calcium plays permissive roles in these physiological processes rather than triggers them; however, the regulation in [Ca^2+^]_i_ levels appears to be important for proper metabolic signaling in mature adipocytes. Moreover it has been reported that intracellular Mg^2+^ deficiency is related to metabolic disorders, such as diabetes mellitus (Resnick et al. 1993; Takaya et al. 2004; Barbagallo and Dominguez 2007). Thus, if it is expressed in adipocytes, TRPM7 might affect the physiological functions through the regulation of [Ca^2+^]_i_ and/or [Mg^2+^]_i_.

Here we demonstrated that TRPM7 was functionally expressed in both freshly isolated mature adipocytes and differentiated 3T3‐L1 adipocytes by patch‐clamp experiments. Consistently, ratiometric Ca^2+^ imaging with Fura‐2 revealed that the augmentation of TRPM7 current by a TRPM7 activator, naltriben (Hofmann et al. 2014), induced an increase in [Ca^2+^]_i_ in differentiated 3T3‐L1 adipocytes.

Portions of this work have appeared previously in abstract form (Inoue et al. 2017).

## Materials and Methods

### Animals

All experimental procedures involving animals were approved in advance by the Institutional Animal Care and Use Committee of Tokyo Medical University and by the Ethics Review Committee for Animal Care and Experimentation of the National Institute for Physiological Sciences. C57BL/6 mice aged 10 weeks were purchased from Japan SLC (Shizuoka, Japan).

### Reagents

2‐aminoethyl diphenylborinate (2‐APB), N‐methyl maleimide (NMM), NS8593, naltriben, dexamethasone (DEX), and 3‐isobuthyl‐1‐methylxanthine (IBMX) were purchased from Sigma‐Aldrich (St. Louis, MO). 2‐amino‐2‐[2‐(4‐octylphenyl)ethyl]‐1,3‐propanediol (FTY720) was purchased from Cayman Chemicals (Ann Arbor, MI). Recombinant human insulin was purchased from Wako (Osaka, Japan). Fura‐2 acetoxymethyl ester (AM) was purchased from Thermo Fisher Scientific (Waltham, MA).

### Preparation of white adipocytes and differentiated 3T3‐L1 cells

Freshly isolated murine white adipocytes were prepared as described previously (Inoue et al. 2010). 3T3‐L1 cells were grown in Dulbecco’s modified Eagle’s medium (DMEM; Sigma‐Aldrich) supplemented with 10% calf serum, GlutaMAX™‐I (Thermo Fisher Scientific), and 1% penicillin‐streptomycin (Thermo Fisher Scientific) at 37°C in 5% CO_2_ under humidified conditions. Three days after confluence, cells were differentiated into adipocytes with a differentiation medium, consisting of DMEM supplemented with 10% fetal bovine serum, 2 mmol/L glutamine, penicillin‐streptomycin, and a differentiation cocktail (0.25 *μ*mol/L DEX, 10 *μ*g/mL insulin, and 0.5 mmol/L IBMX). After 42 h, the medium was changed to a fresh differentiation medium without the differentiation cocktail, and then replaced with fresh differentiation medium every 2 days. Differentiated 3T3‐L1 cells were harvested by trypsin digestion and plated on Matrigel^®^ (Corning, Corning, NY)‐coated glass coverslips for patch‐clamp experiments and Ca^2+^ imaging experiments, or for siRNA transfection.

### siRNA transfection

Transfection of TRPM7‐targeted siRNA into 3T3‐L1 adipocytes was performed as described previously (Kilroy et al. 2009). In a collagen‐coated 48‐well plate (BD, Franklin Lakes, NJ), 10 *μ*L Opti‐MEM (Thermo Fisher Scientific) and 10 *μ*L of 2 *μ*mol/L siRNA (final concentration: 100 nmol/L) were mixed and incubated for 5 min at room temperature. TRPM7‐targeted siRNA and nontargeted siRNA were used (*Silencer* ^®^ Select s81666 and negative control 1 from Thermo Fisher Scientific). Subsequently, 18.6 *μ*L Opti‐MEM and 1.4 *μ*L of a lipid‐based transfection reagent, DharmaFECT 4 (GE Healthcare Life Science, Buckinghamshire, UK), were added and incubated for 20 min at room temperature. A suspension of differentiated 3T3‐L1 adipocytes was then added to the plate containing siRNA complex at a density of 1.02–1.16 × 10^5^ cells/cm^2^ and cultured for 24 h. To visualize cells transfected with siRNA, siGLO Green Transfection Indicator (Thermo Fisher Scientific) was co‐transfected. Twenty‐four hours after transfection, cells were re‐plated onto Matrigel‐coated glass cover slips and then cultured an additional 18 to 24 h before patch‐clamp experiments or Ca^2+^ imaging experiments.

### Quantitative real‐time polymerase chain reaction (PCR)

First‐strand cDNA of 3T3‐L1 adipocytes was prepared using the SuperScript™ III CellsDirect cDNA Synthesis System (Thermo Fisher Scientific) following manufacturer’s instructions. The expression level of TRPM7 was determined by quantitative real‐time polymerase chain reaction (PCR) in each sample using the ABI 7500 Real‐Time PCR system (Thermo Fisher Scientific). The expression level of the house‐keeping gene TATA box binding protein was used as an internal control. Primers and probes used for target genes were TaqMan Gene Expression Assays (Mm00446973_m1 for TATA box‐binding protein; Mm00457998_m1 for TRPM7) purchased from Thermo Fisher Scientific.

### Patch‐clamp experiments

**Figure 1:**
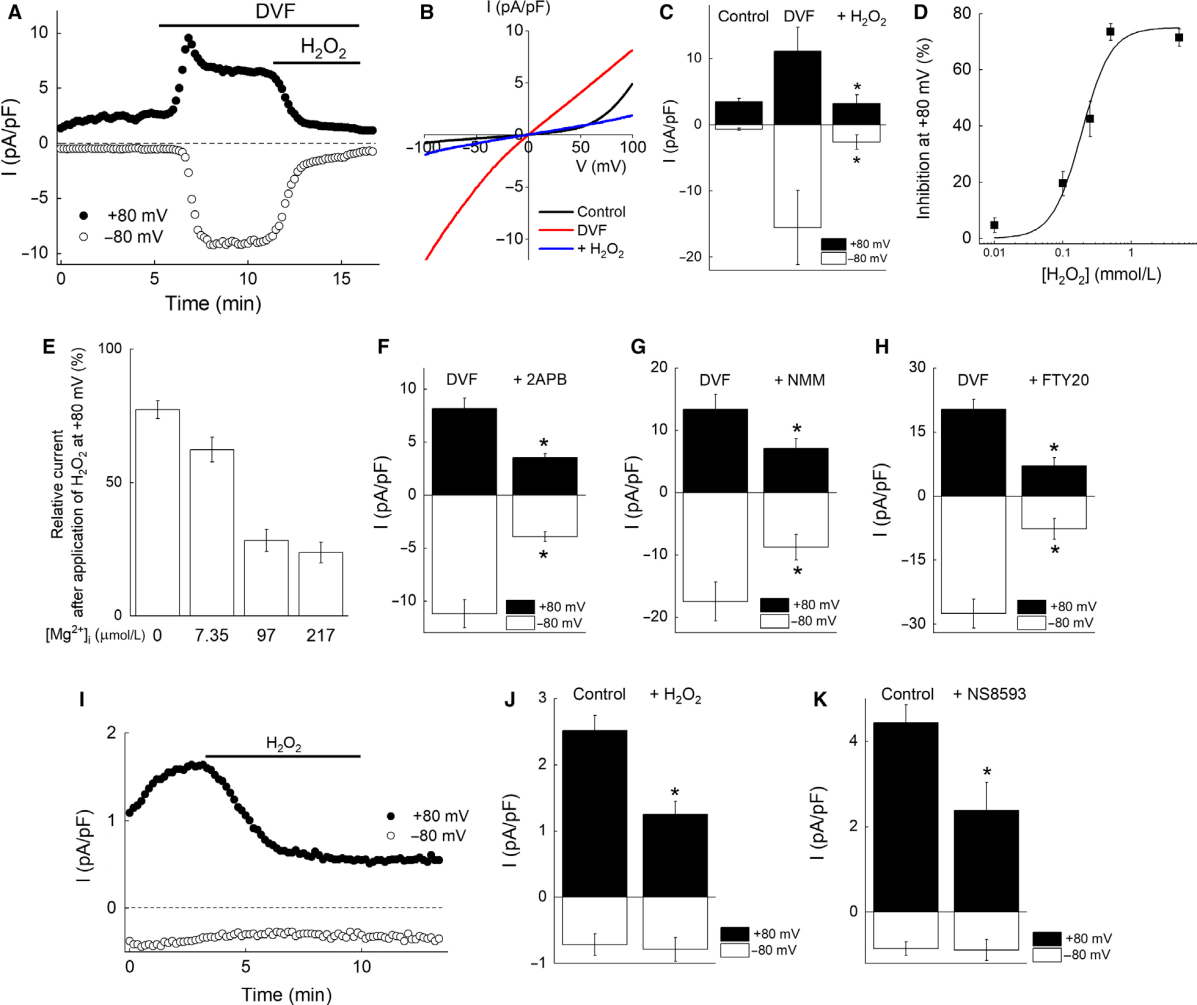
TRPM7‐like currents in native white adipocytes from mice. (A) A representative trace of whole‐cell currents showing the time course of current activation by application of divalent‐free solution (DVF) and inhibition by the addition of H_2_O_2_ (500 *μ*mol/L) in the presence of 29 *μ*mol/L [Mg^2+^]_i_ in a native adipocyte. Ramp command pulses from −100 to +100 mV (1 sec duration) were applied every 10 sec, and the current amplitude at +80 mV (closed circles) or −80 mV (open circles) was plotted against the recording time. (B) Representative *I–V* relationship of TRPM7‐like current recorded from the same cell shown in A under the control (black line), DVF (red line), or 4 min after application of H_2_O_2_ (500 *μ*mol/L) (blue line). (C) DVF induced a substantial increase in both inward and outward currents in native adipocytes. Each bar represents the mean ± SEM (vertical bar) of four observations. *P < 0.05 versus DVF. (D) H_2_O_2_ inhibited the current in a concentration‐dependent manner with IC_50_ at 187 *μ*mol/L. (E) Inhibition of TRPM7‐like current was dependent on [Mg^2+^]_i_. Each bar represents the mean ± SEM (vertical bar) of the current density at +80 mV after the application of H_2_O_2_ (500 *μ*mol/L) relative to that before application (5–9 observations). (F, G, H) Effect of 2‐APB (200 *μ*mol/L) (F), NMM (50 *μ*mol/L) (G), and FTY720 (10 *μ*mol/L) (H) on the DVF‐activated, TRPM7‐like current in native white adipocytes. Each bar represents the mean ± SEM (vertical bar) of eight observations. *P < 0.05 versus DVF. (I) A representative trace of whole‐cell currents showing the time course of current inhibition by an addition of H_2_O_2_ (500 *μ*mol/L) in the presence of extracellular divalent cations in native adipocytes. (J) Effect of H_2_O_2_ (500 *μ*mol/L) on the basal TRPM7‐like current in native adipocytes. Each bar represents the mean ± SEM (vertical bar) of 8 observations. *P < 0.05 versus control. (K) Effect of NS8593 (20 *μ*mol/L) on the basal TRPM7‐like current in native adipocytes. Each bar represents the mean ± SEM (vertical bar) of five observations. *P < 0.05 versus control.

All experiments were conducted at room temperature (23–25°C). A salt bridge containing 3 mol/L KCl in 2% agarose was used to connect a reference Ag–AgCl electrode. The patch electrodes, prepared from borosilicate glass capillaries, had a resistance of 1.5–2.5 MΩ when filled with a pipette solution (see below). Series resistance (<5 MΩ) was compensated to 80% to minimize voltage errors. Currents were recorded using an Axopatch 200B amplifier (Molecular Devices, San Jose, CA) coupled to a DigiData 1321A A/D and D/A converter (Molecular Devices). Current signals were filtered at 1 kHz using a four‐pole Bessel filter and were digitized at 5 kHz. pClamp 10.4 software was used for the command pulse protocol, data acquisition, and analysis. The time courses of current activation and recovery were monitored by repetitively applying (every 10 sec) ramp pulses from −100 to +100 mV (1 sec duration) from a holding potential of 0 mV. The control bath solution consisted of (mmol/L): 135 NaCl, 5 KCl, 1 MgCl_2_, 1 CaCl_2_, 1.2 NaH_2_PO_4_, 10 HEPES, 2 glucose, and 27 mannitol (pH 7.4 adjusted with NaOH, 315 mOsmol/kgH_2_O). Divalent‐free bath solution (DVF) was made by omitting MgCl_2_ and CaCl_2_, and adding 0.5 mmol/L EDTA and 0.2 mmol/L EGTA. The intracellular (pipette) solutions were as follows (mmol/L): 25 CsCl, 135 CsOH, 135 glutamate, 5 HEPES, 1 MgCl_2_, 5 Na_2_ATP, and 0.1 NaGTP (pH 7.3 adjusted with CsOH, 290 mOsmol/kgH_2_O, free [Mg^2+^]_i_ = 29 *μ*mol/L). When [Mg^2+^]_i_ was varied to 0, 7.35, 97, and 217 *μ*mol/L (Fig. 1D), the pipette solutions were as follows (mmol/L): 25 CsCl, 110 CsOH, 110 glutamate, 0.2 EGTA, 10 EDTA (or HEDTA), 5 HEPES (pH 7.3 adjusted with CsOH, 290 mOsmol/kgH_2_O). MgSO_4_ was added to vary [Mg^2+^]_i_. [Mg^2+^] was calculated by Maxchelator software (https://somapp.ucdmc.ucdavis.edu/pharmacology/bers/maxchelator/).

### Ca^2+^ imaging experiments

Differentiated 3T3‐L1 adipocytes were loaded with 1 *μ*mol/L Fura‐2 AM for 30 min at 37°C in HEPES‐buffered saline (HBS) consisting of (mmol/L): 135 NaCl, 5 KCl, 2 CaCl_2_, 1.2 NaH_2_PO_4_, 10 HEPES, and 2 glucose (pH 7.4 adjusted with NaOH). After loading, cells were superfused with HBS for 15 min at room temperature. Calcium‐free HBS was made by omitting CaCl_2_ and adding 0.2 mmol/L EGTA. Fluorescent images of Fura‐2 excited at 340 and 380 nm were acquired using a scientific CMOS camera (ORCA‐Flash4.0, Hamamatsu Photonics, Shizuoka, Japan) with 40× objective (UPlanSApo, numerical aperture of 0.95, Olympus, Tokyo, Japan) of an inverted microscope (IX83, Olympus). Data were collected and analyzed with image analysis software (cellSens, Olympus). [Ca^2+^]_i_ was monitored by fluorescence ratio (F340/F380).

### Statistical analysis

**Figure 2:**
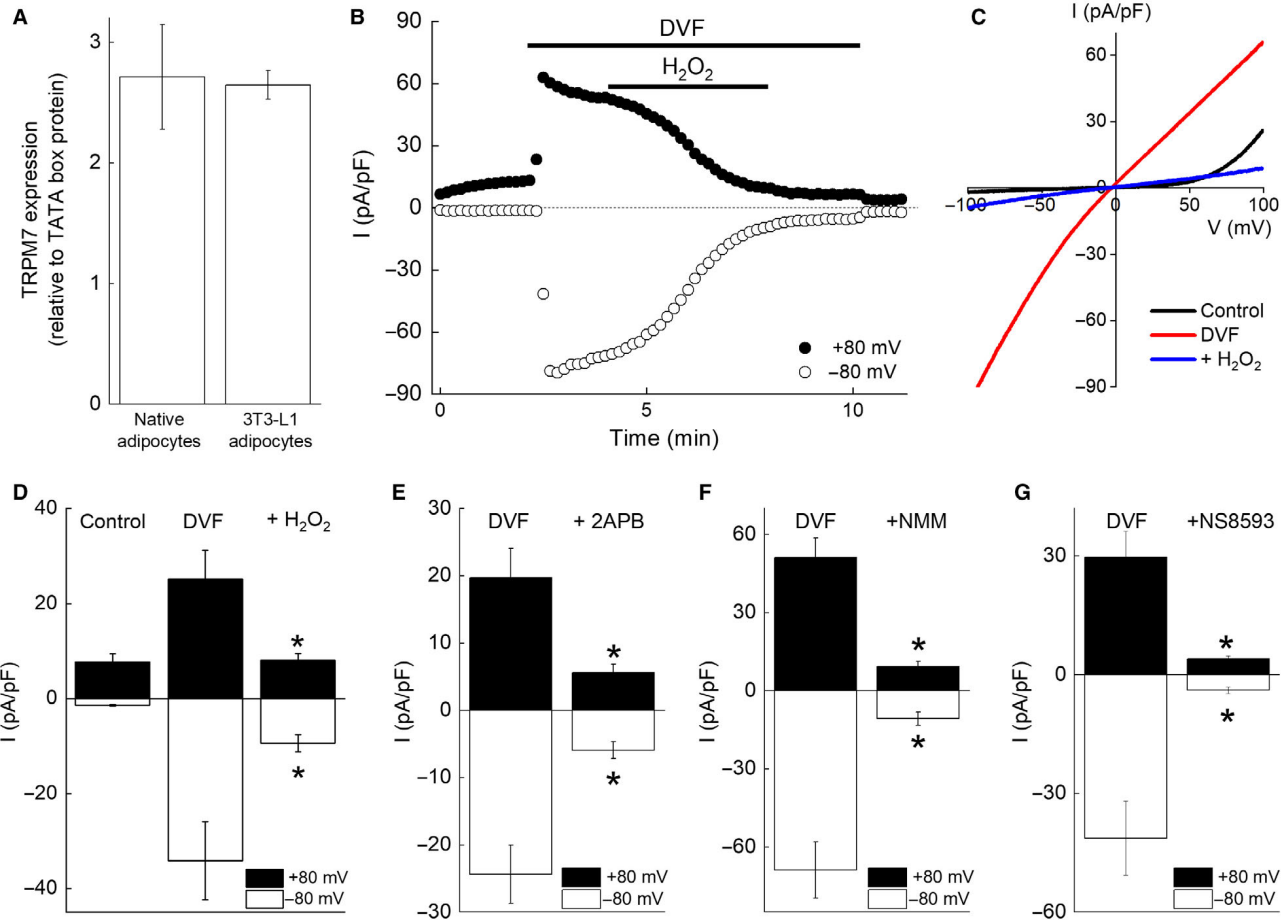
TRPM7‐like currents in differentiated 3T3‐L1 adipocytes. (A) The expression of TRPM7 mRNA in native white adipocytes and 3T3‐L1 adipocytes. Each bar represents the mean ± SEM (vertical bar) of 4 to 5 observations. (B) A representative trace of whole‐cell currents showing the time course of current activation by an application of divalent‐free solution (DVF) and inhibition by the addition of H_2_O_2_ (500 *μ*mol/L) in a differentiated 3T3‐L1 adipocyte in the presence of 29 *μ*mol/L [Mg^2+^]_i_. The current amplitude at +80 mV (closed circles) or −80 mV (open circles) was plotted against the recording time. (C) Representative *I*–*V* relationship of TRPM7‐like current recorded from the same cell shown in B under the control (black line), DVF (red line), or 4 min after application of H_2_O_2_ (500 *μ*mol/L) (blue line). (D) DVF induced a substantial increase in both inward and outward currents in 3T3‐L1 adipocytes. Each bar represents the mean ± SEM (vertical bar) of 9 observations. *P < 0.05 versus DVF. (E–G) Effect of 2‐APB (200 *μ*mol/L) (E), NMM (100 *μ*mol/L) (F), and NS8593 (20 *μ*mol/L) (G) on the DVF‐activated, TRPM7‐like current in 3T3‐L1 adipocytes. Each bar represents the mean ± SEM (vertical bar) of 6 to 8 observations. *P < 0.05 versus DVF.

**Figure 3:**
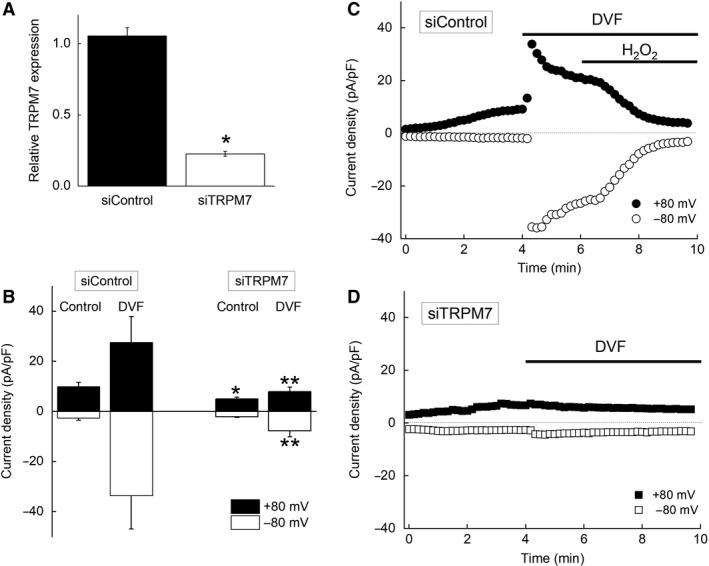
Effects of TRPM7‐knockdown by siRNA in differentiated 3T3‐L1 adipocytes. (A) The relative expression of mRNA of TRPM7 in siControl‐ and siTRPM7‐adipocytes. Each bar represents the mean ± SEM (vertical bar) of 19 observations. *P < 0.05 versus siControl‐adipocytes. (B) Divalent‐free solution (DVF) induced a substantial increase in both inward and outward currents in siControl‐adipocytes, but not in siTRPM7‐adipocytes. Each bar represents the mean ± SEM (vertical bar) of 4 to 5 observations. *P < 0.05 versus control in siControl‐adipocytes. **P < 0.05 versus DVF in siControl‐adipocytes. (C and D) Representative traces of whole‐cell currents showing the time course of current activation by an application of DVF and inhibition by an addition of H_2_O_2_ (500 *μ*mol/L) in a siControl‐adipocyte (C) or a siTRPM7‐adipocyte (D) in the presence of 29 *μ*mol/L [Mg^2+^]_i_. The current amplitude at +80 mV (closed circles) or −80 mV (open circles) was plotted against the recording time.

**Figure 4:**
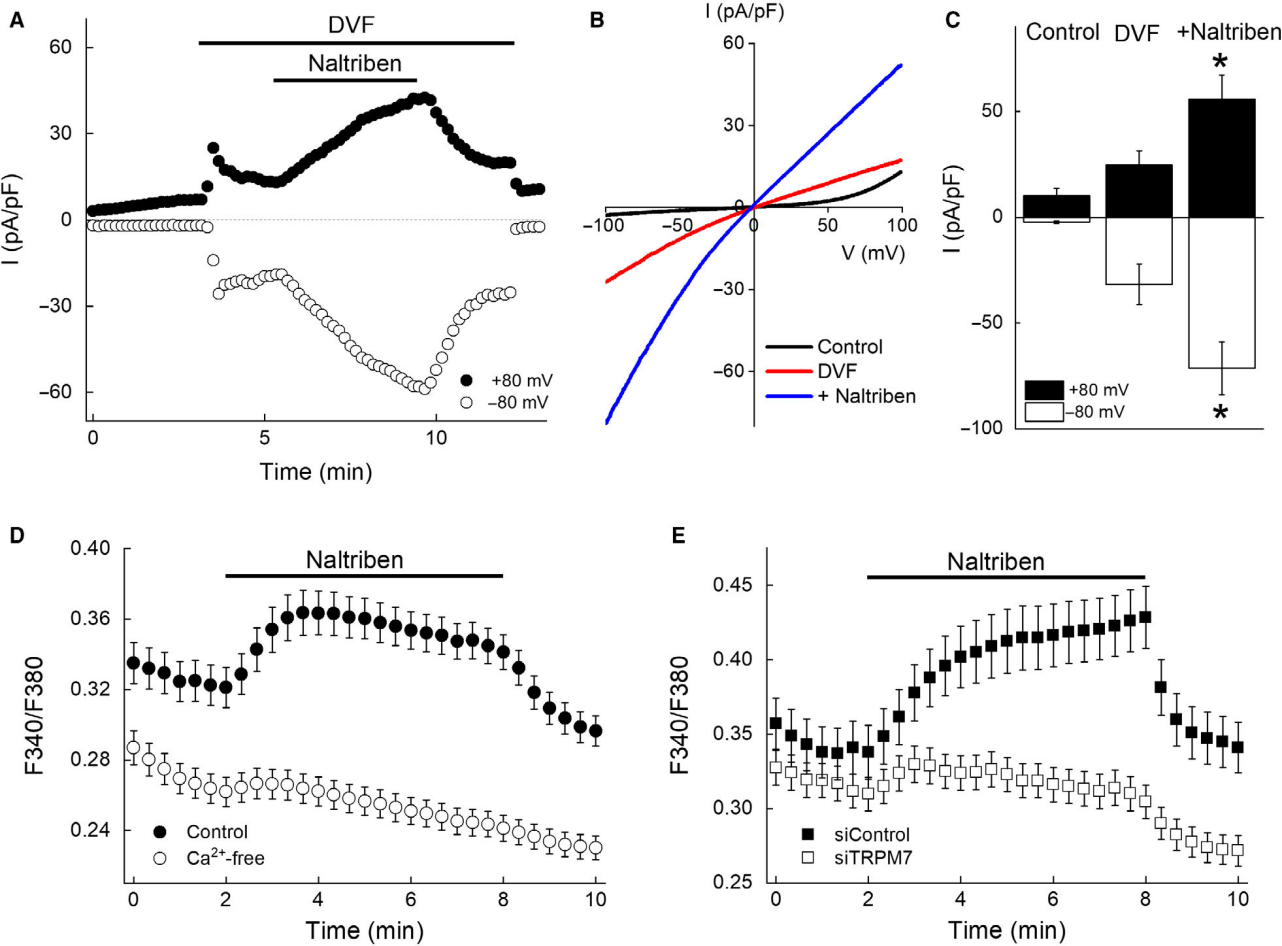
Effects of naltriben on TRPM7 current and [Ca^2+^]_i_ in 3T3‐L1 adipocytes. (A) A representative trace of whole‐cell currents showing the time course of current activation by an application of DVF and augmentation by the addition of naltriben (50 *μ*mol/L) in a differentiated 3T3‐L1 adipocyte in the presence of 29 *μ*mol/L [Mg^2+^]_i_. The current amplitude at +80 mV (closed circles) or −80 mV (open circles) was plotted against the recording time. (B) Representative *I–V* relationship of TRPM7 current recorded from the same cell shown in A under the control (black line), DVF (red line), or 4 min after application of naltriben (50 *μ*mol/L) (blue line). (C) Both inward and outward currents were augmented in 3T3‐L1 adipocytes. Each bar represents the mean ± SEM (vertical bar) of six observations. *P < 0.05 versus DVF. (D) Ratiometric Ca^2+^ imaging in 3T3‐L1 adipocytes. Naltriben (50 *μ*mol/L) increased [Ca^2+^]_i_, as indicated by the fluorescence ratio (F340/F380), in the presence of 2 mmol/L [Ca^2+^]_o_, but not in the absence of extracellular Ca^2+^. Each symbol represents the mean ± SEM (vertical bar) of 66 or 104 observations, in control or Ca^2+^‐free conditions, respectively. (E) Naltriben (50 *μ*mol/L) increased [Ca^2+^]_i_ in siControl‐adipocytes (closed squares) but not in siTRPM7‐adipocytes (open squares). Each symbol represents the mean ± SEM (vertical bar) of 56 or 50 observations, in siControl‐adipocytes or siTRPM7‐adipocytes, respectively.

Data are shown as the mean ± standard error of the mean (SEM) of observations. Statistical analysis was performed between the two groups using a paired *t*‐test (Figs. 1F–H, J and K and 2E–G) or an unpaired *t*‐test (Fig. 3A and B). A one‐way analysis of variance (ANOVA) followed by post hoc Bonferroni test was used to analyze the data of Figures 1C, 2D, and 4C. Data were considered to be significant at *P* < 0.05.

## Results

### TRPM7‐like current in acutely isolated mouse white adipocytes

Whole‐cell patch‐clamp recordings were performed to investigate whether native mouse white adipocytes functionally express TRPM7 current. It has been reported that TRPM7 is (1) inhibited by intracellular Mg^2+^ (Nadler et al. 2001; Schmitz et al. 2003; Chokshi et al. 2012b,2012c); (2) activated by the elimination of divalent cations from the bath solutions, resulting in quasi‐linear current‐voltage (*I*–*V*) relationships (Nadler et al. 2001; Kerschbaum et al. 2003; Inoue et al. 2014); (3) inhibited by H_2_O_2_ irreversibly in a [Mg^2+^]_i_‐dependent manner (Inoue et al. 2014); and (4) inhibited by 2‐aminoethyl diphenylborinate (2‐APB) (Li et al. 2006; Macianskiene et al. 2012; Chokshi et al. 2012a), N‐methyl maleimide (NMM) (Inoue et al. 2014), NS8593 (Chubanov et al. 2012; Tashiro et al. 2014, 2019), and FTY720 (Qin et al. 2013; Takahashi et al. 2018).

Whole‐cell recordings in native white adipocytes revealed that outwardly rectifying currents were gradually activated after the establishment of whole‐cell configurations with the pipette solution that contained 29 *μ*mol/L [Mg^2+^] in control bath solution (Fig. 1A). As TRPM7 current is inhibited by intracellular free Mg^2+^ (normally ~0.9 mmol/L in cytosol), these currents may contain TRPM7 current, which is activated due to a reduction in intracellular Mg^2+^ concentration to 29 *μ*mol/L by intracellular perfusion with the pipette solution (Inoue et al. 2014). The current was further activated by perfusion with DVF in native white adipocytes (3.5 ± 0.5 and −0.7 ± 0.2 pA/pF in the control solution vs. 11.1 ± 3.6 and −15.5 ± 5.9 pA/pF in DVF at +80 and −80 mV [*n* = 4], respectively) (Fig. 1A and B). H_2_O_2_ inhibited the DVF‐activated current in a concentration‐dependent manner with half‐maximal inhibition concentration (IC_50_) of 187 *μ*mol/L (Fig. 1D). At 500 *μ*mol/L, H_2_O_2_ maximally inhibited the current (Fig. 1A–D). The inhibition by H_2_O_2_ was dependent on [Mg^2+^]_i_ (Fig. 1E). Relative currents after the application of H_2_O_2_ were 77.3 ± 3.4, 62.3 ± 4.7, 28.3 ± 4.2, and 23.8 ± 3.9% in the absence or presence of 7.35, 97, and 217 *μ*mol/L [Mg^2+^]_i_, respectively (Fig. 1D). Application of 2‐APB (200 *μ*mol/L), NMM (50 *μ*mol/L), or FTY720 (10 *μ*mol/L) inhibited the DVF‐activated current in white adipocytes (Fig. 1F–H). Moreover the outward current in the presence of extracellular divalent cations was also inhibited by H_2_O_2_ (500 *μ*mol/L) as well as NS8593 (20 *μ*mol/L), which has been reported to inhibit TRPM7 current at low concentrations with an IC_50_ of 1.6 *μ*mol/L (Chubanov et al. 2012), though the inward current was too small to detect the significant inhibitory effects of these reagents (Fig. 1I–K). These electrophysiology results suggest the existence of TRPM7‐like current in white adipocytes.

### TRPM7‐like current detected in differentiated 3T3‐L1 adipocytes

3T3‐L1 is a widely used cell line that can differentiate into lipid‐filled adipocytes in culture. To explore whether differentiated 3T3‐L1 adipocytes expressed TRPM7 comparable to native white adipocytes, TRPM7 mRNA expression was compared by quantitative real‐time PCR. The expression level of TRPM7 mRNA in differentiated 3T3‐L1 adipocytes was similar to that in native white adipocytes (Fig. 2A). Consistently, similar TRPM7‐like current was activated by the extracellular perfusion of DVF and inhibited by H_2_O_2_ (500 *μ*mol/L), 2‐APB (200 *μ*mol/L), NMM (100 *μ*mol/L), and NS8593 (20 *μ*mol/L) in differentiated 3T3‐L1 adipocytes compared to native white adipocytes (Fig. 2B–G). These results suggest that the DVF‐activated current is TRPM7 current in acutely isolated native white adipocytes and in differentiated 3T3‐L1 adipocytes.

### TRPM7‐like current was reduced in differentiated 3T3‐L1 adipocytes transfected with TRPM7‐targeted siRNA

Transfection of differentiated 3T3‐L1 adipocytes with siRNA targeting TRPM7 (siTRPM7) resulted in the reduction of TRPM7 mRNA expression to 22.6 ± 1.7% of nontargeting siRNA (siControl) at post‐transfection day 2 (Fig. 3A). To explore the effect of siRNA introduction on TRPM7‐like current, whole‐cell currents were recorded in differentiated 3T3‐L1 adipocytes. Transfected cells were visualized by co‐transfection with siRNA and siGLO green. Both the control currents (10.0 ± 1.7 and −2.6 ± 1.0 pA/pF in siControl‐adipocytes [*n* = 5] vs. 5.1 ± 0.7 and −2.1 ± 0.3 pA/pF in siTRPM7‐adipocytes [*n* = 6] at +80 and −80 mV, respectively) and the DVF‐activated currents were dramatically reduced in siTRPM7‐transfected adipocytes compared to siControl‐transfected adipocytes (27.5 ± 10.4 and −33.6 ± 13.4 pA/pF in siControl‐adipocytes [*n* = 4] vs. 7.9 ± 1.8 and −7.7 ± 2.4 pA/pF in siTRPM7‐adipocytes [*n* = 6] at +80 and −80 mV, respectively) (Fig. 3B–D).

### Naltriben activated TRPM7 current and increased [Ca^2+^]_i_ in differentiated 3T3‐L1 adipocytes

Naltriben has been reported to activate TRPM7 (Hofmann et al. 2014; Wong et al. 2017; Tashiro et al. 2019). Consistently, the DVF‐activated TRPM7 current was increased ~2.5‐fold by an application of naltriben (50 *μ*mol/L) in differentiated 3T3‐L1 adipocytes (Fig. 4A–C). TRPM7 current was increased gradually during a 4 min application of naltriben (24.7 ± 6.7 and −31.7 ± 9.6 pA/pF in DVF vs. 55.8 ± 11.4 and −71.4 ± 12.5 pA/pF in the presence of naltriben [*n* = 6] at + 80 mV and −80 mV, respectively), and was reversed to the levels before application (Fig. 4A). Moreover [Ca^2+^]_i_ levels were also increased by an application of naltriben (50 *μ*mol/L) in the presence of 2 mmol/L [Ca^2+^]_o_ (Fig. 4D). [Ca^2+^]_i_ was reversed by the elimination of naltriben from the extracellular solution. Naltriben failed to increase [Ca^2+^]_i_ in the absence of extracellular Ca^2+^ (Fig. 4D). TRPM7‐knockdown significantly impaired naltriben‐induced [Ca^2+^]_i_ increase. The changes in the Fura‐2 ratio at 6 min after an application of naltriben were 0.091 ± 0.011 in siControl‐adipocyte (*n* = 56) and −0.005 ± 0.006 in siTRPM7‐adipocyte (*n* = 50), but the resting levels of [Ca^2+^]_i_ did not differ between siControl‐adipocytes and siTRPM7‐adipocytes (Fig. 4E). These data suggest that the naltriben‐induced [Ca^2+^]_i_ increase was due to Ca^2+^ influx from the extracellular space via TRPM7.

## Discussion

Adipocytes secrete various hormone‐like factors, the so‐called adipokines. It has been suggested that Ca^2+^ modulates intracellular signaling, which mediates the secretion of adipokines (Levy et al. 2000; Cammisotto and Bukowiecki 2004; Sukumar et al. 2012; Komai et al. 2014; Schlottmann et al. 2014; El Hachmane et al. 2015) and the insulin response (Draznin et al. 1987; Kelly et al. 1989; Whitehead et al. 2001; Worrall and Olefsky 2002). It has been reported that there are several functional Ca^2+^ influx pathways, including VDCC (Draznin et al. 1987; Pershadsingh et al. 1989; Levy et al. 2000), TRPC1/5 (Sukumar et al. 2012; Bishnoi et al. 2013; El Hachmane and Olofsson 2018), TRPV4 (Ye et al. 2012; Bishnoi et al. 2013; Sanchez et al. 2016), and STIM1/ORAI1 (El Hachmane et al. 2018), in adipocytes. In the present study, we found that TRPM7, a Ca^2+^/Mg^2+^‐permeable cation channel, is functionally expressed in both native white adipocytes and differentiated 3T3‐L1 adipocytes, and plays a role as a Ca^2+^ influx pathway.

The characteristics of the DVF‐activated, TRPM7‐like current in native white adipocytes and differentiated 3T3‐L1 adipocytes were quite consistent with those reported for heterologously overexpressed TRPM7 current. TRPM7‐like currents in both cell types were activated by a reduction of [Mg^2+^]_i_, an elimination of extracellular divalent cations, and were inhibited by 2‐APB, NS8593, and FTY720 (Figs. 1 and 2), which were consistent with previous reports (Nadler et al. 2001; Schmitz et al. 2003; Chubanov et al. 2012; Chokshi et al. 2012b,2012c; Qin et al. 2013; Tashiro et al. 2014, 2019). We previously reported that TRPM7 current was inhibited by H_2_O_2_ in a [Mg^2+^]_i_‐ and [ATP]_i_‐dependent manner in HEK293 cells overexpressing murine TRPM7 (Inoue et al. 2014). In the presence of 5 mmol/L [ATP]_i_, H_2_O_2_ failed to inhibit overexpressed TRPM7 current (Inoue et al. 2014). In the present study, the endogenous TRPM7‐like current was inhibited by H_2_O_2_ in a [Mg^2+^]_i_‐dependent manner; however, the inclusion of 5 mmol/L [ATP]_i_ did not affect the current inhibition by H_2_O_2_ (Figs. 1A–D, I and J, 2B–D, 3C). Although it remains unclear why the effect of ATP differs between endogenous TRPM7‐like current and overexpressed TRPM7 current, based on overall similarities in their characteristics and the observation that siTRPM7 effectively reduced the current, our data suggest that TRPM7 is functionally expressed in native adipocytes and in differentiated 3T3‐L1 adipocytes.

Naltriben augmented Ca^2+^ influx as well as the current (Fig. 4), indicating that TRPM7 is one of the Ca^2+^ influx pathways in adipocytes. Although the current recording showed the growing current during a 4 min application of naltriben (Fig. 4A), the increase in [Ca^2+^]_i_ reached a plateau within 2 min (Fig. 4D). This might result from the inhibition of the channel by intracellular Ca^2+^ (Kozak et al. 2005; Matsushita et al. 2005) that did not occur during the whole‐cell path‐clamp recordings due to intracellular perfusion by the pipette solution, and/or the activity of Ca^2+^ extrusion by calmodulin‐sensitive Ca^2+^ pumps (Pershadsingh et al. 1980) and Na^+^/Ca^2+^ exchanger (Pershadsingh et al. 1989) in adipocytes. Since [Ca^2+^]_i_ is finely tuned by multiple molecules, TRPM7‐knockdown did not significantly affect resting [Ca^2+^]_i_ in 3T3‐L1 adipocytes (Fig. 4E). In contrast, the naltriben‐stimulated [Ca^2+^]_i_ increase was clearly observed in siControl‐adipocytes but not in siTRPM7‐adipocytes. Thus, Ca^2+^ influx via TRPM7 might exert physiological effects, especially when the TRPM7 channel is activated.

It is well established that TRPM7 channel activity is tonically regulated by intracellular free Mg^2+^ as well as Mg‐nucleotides (Penner and Fleig 2007); however, little is known about the physiological stimuli that change the TRPM7 channel activity. Several reports indicate that activation of phospholipase C (PLC) induces Ca^2+^ influx via TRPM7 in intact cells (Langeslag et al. 2007; Qian et al. 2019). There are contradicting reports showing that TRPM7 is inactivated by G_αq_‐coupled receptor stimulation via the PLC‐mediating depletion of phosphatidylinositol 4,5‐bisphosphates (PIP_2_) which is necessary for TRPM7 channel activity (Runnels et al. 2002; Kozak et al. 2005; Gwanyanya et al. 2006; Bates‐Withers et al. 2011; Zhelay et al. 2018). Therefore, the physiological stimuli that activate PLC in adipocytes might increase or decrease [Ca^2+^]_i_ by activation or inactivation of TRPM7. Insulin has been reported to activate PLC in adipocytes; however it remains controversial whether insulin induces Ca^2+^ flux (Draznin et al. 1987; Cammisotto and Bukowiecki 2004). It might be possible that very local changes of [Ca^2+^] near TRPM7 channel can be induced by insulin via PLC in adipocytes. Further study is needed to clarify if there are physiological stimuli that cause Ca^2+^ influx via TRPM7 in adipocytes.

In pre‐adipocytes, it has been reported that TRPM7 is functionally expressed and involved in proliferation and adipogenesis (Chen et al. 2014). Consistently, we examined TRPM7 mRNA expression in 3T3‐L1 pre‐adipocytes and found that the expression levels of TRPM7 were comparable between pre‐adipocytes and differentiated 3T3‐L1 adipocytes (data not shown). Thus, unlike Cav3.1, which is downregulated in mature adipocytes (Oguri et al. 2010), or TRPC1/C5, which are upregulated by differentiation (Sukumar et al. 2012), TRPM7 was expressed constantly irrespective of differentiation in adipose cells. The physiological roles of TRPM7 in mature adipocytes remain unknown. Since TRPM7 is a Ca^2+^/Mg^2+^‐permeable channel that contains a protein kinase domain, it likely exerts functions via Ca^2+^/Mg^2+^ influx and/or its kinase activity. In adipocytes, Ca^2+^ modulates lipolysis (Tebar et al. 1996; Xue et al. 1998), glucose uptake (Draznin et al. 1987; Kelly et al. 1989; Whitehead et al. 2001; Worrall and Olefsky 2002), and the secretion of adipokines (Levy et al. 2000; Cammisotto and Bukowiecki 2004; Sukumar et al. 2012; Komai et al. 2014; Schlottmann et al. 2014; El Hachmane et al. 2015). It has been reported that the reduction in intracellular Mg^2+^ content is related to impaired glucose metabolism and insulin resistance (Kandeel et al. 1996; Takaya et al. 2004; Barbagallo and Dominguez 2007). One of the reported substrates of TRPM7 kinase, myosin IIA, is involved in glucose transporter 4 translocation upon insulin stimulation (Steimle et al. 2005; Fulcher et al. 2008; Chung et al. 2010; Stall et al. 2014). Phosphorylation of myosin IIA by TRPM7 inhibits myosin II‐based contractility (Clark et al. 2006, 2008). Thus, it is likely that downregulation of TRPM7 affects these functions. In our pilot studies investigating the effects of TRPM7‐knockdown on lipolysis and insulin‐stimulated glucose uptake, there was no significant difference between siControl and siTRPM7 (data not shown). Furthermore, released adipokines in culture supernatants were roughly compared using a Profiler Mouse Adipokine Array (R&D Systems, Minneapolis, MN), and the results revealed that there was no marked difference between siControl and siTRPM7 in our current preparations (~65% knockdown of TRPM7 in 3T3‐L1 adipocytes differentiated for 9 days in culture). Thus, further study is needed to understand the physiological roles of TRPM7 in adipocytes under diverse conditions (e.g., using TRPM7‐deficient or overexpressing matured adipocytes, and adipocyte‐specific TRPM7‐deficient mice).

In conclusion, we determined the functional expression of TRPM7 by whole‐cell path‐clamp and Ca^2+^ imaging experiments in adipocytes. TRPM7 may modulate physiological functions of adipocytes by permeating Ca^2+^/Mg^2+^ and/or phosphorylating the substrates.

## Conflict of Interest

No conflicts of interest are declared by the authors.
